# Predictors of Chikungunya rheumatism: a prognostic survey ancillary to the TELECHIK cohort study

**DOI:** 10.1186/ar4137

**Published:** 2013-01-09

**Authors:** Patrick Gérardin, Adrian Fianu, Alain Michault, Corinne Mussard, Karim Boussaïd, Olivier Rollot, Philippe Grivard, Somar Kassab, Eric Bouquillard, Gianandrea Borgherini, Bernard-Alex Gaüzère, Denis Malvy, Gérard Bréart, François Favier

**Affiliations:** 1 Centre for Clinical Investigation-Clinical Epidemiology (CIC-EC) of La Réunion (INSERM/CHU/Université de La Réunion/URMLR-OI), Groupe Hospitalier Sud Réunion, Avenue François Mitterrand, 97448 Saint Pierre cedex, La Réunion, France; 2 Neonatal and Paediatric Intensive Care Unit, CHU, Groupe Hospitalier Sud Réunion, Avenue François Mitterrand, 97448 Saint Pierre cedex, La Réunion, France; 3 UMRS 953, "Epidemiological Research Unit on Perinatal Health and Women and Children Health" (INSERM/Assistance Publique des Hôpitaux de Paris), Maternité de Port Royal, 53, avenue de l'Observatoire, 75014 Paris, France; 4 Bacteriology - Virology - Parasitology - Hygiene, CHU, Groupe Hospitalier Sud Réunion, Avenue François Mitterrand, 97448 Saint Pierre cedex, La Réunion, France; 5 Virology Unit, CHU, Hôpital Pellegrin, Place Amélie Raba Léon, 33076 Bordeaux cedex, France; 6 Rheumatology Clinic, Neurology, CHU, Groupe Hospitalier Sud Réunion, Avenue François Mitterrand, 97448 Saint Pierre cedex, La Réunion, France; 7 Infectious Diseases, CHU, Groupe Hospitalier Sud Réunion, Avenue François Mitterrand, 97448 Saint Pierre cedex, La Réunion, France; 8 Polyvalent Intensive Care Unit, CHU, Centre Hospitalier Félix Guyon, allée des Topazes, 97400 Saint Denis, La Réunion, France; 9 Internal Medicine and Tropical Diseases; Centre of Tropical Medicine René Labusquière, CHU, Hôpital Saint André, 1, rue Jean Burguet, 33075 Bordeaux cedex, France; 10 U897, "Epidemiology and Biostatistics", Institute of Public Health, Epidemiology and Development (INSERM/CHU/Université de Bordeaux 2 Victor Ségalen), 146, rue Léon Saignat, 33076 Bordeaux cedex, France

## Abstract

**Introduction:**

Long-lasting relapsing or lingering rheumatic musculoskeletal pain (RMSP) is the hallmark of Chikungunya virus (CHIKV) rheumatism (CHIK-R). Little is known on their prognostic factors. The aim of this prognostic study was to search the determinants of lingering or relapsing RMSP indicative of CHIK-R.

**Methods:**

Three hundred and forty-six infected adults (age ≥ 15 years) having declared RMSP at disease onset were extracted from the TELECHIK cohort study, Reunion island, and analyzed using a multinomial logistic regression model. We also searched for the predictors of CHIKV-specific IgG titres, assessed at the time of a serosurvey, using multiple linear regression analysis.

**Results:**

Of these, 111 (32.1%) reported relapsing RMSP, 150 (43.3%) lingering RMSP, and 85 (24.6%) had fully recovered (reference group) on average two years after acute infection. In the final model controlling for gender, the determinants of relapsing RMSP were the age 45-59 years (adjusted OR: 2.9, 95% CI: 1.0, 8.6) or greater or equal than 60 years (adjusted OR: 10.4, 95% CI: 3.5, 31.1), severe rheumatic involvement (fever, at least six joints plus four other symptoms) at presentation (adjusted OR: 3.6, 95% CI: 1.5, 8.2), and CHIKV-specific IgG titres (adjusted OR: 3.2, 95% CI: 1.8, 5.5, per one unit increase). Prognostic factors for lingering RMSP were age 45-59 years (adjusted OR: 6.4, 95% CI: 1.8, 22.1) or greater or equal than 60 years (adjusted OR: 22.3, 95% CI: 6.3, 78.1), severe initial rheumatic involvement (adjusted OR: 5.5, 95% CI: 2.2, 13.8) and CHIKV-specific IgG titres (adjusted OR: 6.2, 95% CI: 2.8, 13.2, per one unit increase). CHIKV specific IgG titres were positively correlated with age, female gender and the severity of initial rheumatic symptoms.

**Conclusions:**

Our data support the roles of age, severity at presentation and CHIKV specific IgG titres for predicting CHIK-R. By identifying the prognostic value of the humoral immune response of the host, this work also suggest a significant contribution of the adaptive immune response to the physiopathology of CHIK-R and should help to reconsider the paradigm of this chronic infection primarily shifted towards the involvement of the innate immune response.

## Introduction

Chikungunya virus (CHIKV) is an enveloped RNA positive-strand alphavirus, transmitted by *Aedes *mosquitoes, belonging to the *Togaviridae *family and to the arthritogenic Semliki forest virus (SFV) serocomplex, which includes the well-studied Ross River virus (RRV) [1]. CHIKV is now recognised to target human epithelial and endothelial cells, fibroblasts, dendritic cells, B cells and macrophages [2,3], as well as human muscle satellite cells [4].

The virus is known to cause a wide range of acute manifestations combining fever, arthritis, arthralgias, myalgias, rash and fatigue [5,6]. Furthermore, CHIKV infection often leads to prolonged joint pain, which may harbour two different tempos; either a continuous burden or relapse attacks that characterize the hallmark of Chikungunya rheumatism (CHIK-R) [6-9]. CHIKV might also trigger or reveal inflammatory rheumatic diseases (IRDs) such as rheumatoid-like arthritis (RA) or psoriatic arthritis (PsA) [10,11]. In this context, routine biomarkers (C-reactive protein, CRP; erythrocyte sedimentation rate, ESR), rheumatoid factor, anti-cyclic citrullinated peptide antibodies, HLA-B27 expression, imaging features, or the need for upgraded treatment, usually enable accurate diagnosis. However, for the few CHIK-R patients matching the criteria for IRDs, the differential diagnosis and decision-making for treatment might be even more challenging [12-14].

Of note, the pathomechanisms underlying musculoskeletal pain and chronic arthritide after acute CHIKV infection are partially elucidated. It has been hypothesised that these symptoms may come from the early escape of CHIKV from blood monocytes [3] and its subsequent relocation and persistence in synovial macrophages rather than to an autoimmune process [15] as previously observed with RRV infection [16]. This hypothesis is supported by a recent macaque model in which Chikungunya disease involves long-term viral persistence in macrophages of lymphoid, liver, joint and muscle tissues [17]. Moreover, a positive association between high titres of CHIKV-specific IgG antibodies and long-lasting arthralgias has been observed contemporaneously from a pilot study by our group, and in an Italian cohort, without the pathophysiological significance or clinical impact of such relationship having been sought [18,19].

To date, the predictors of the course of CHIK-R have so far been poorly investigated [8]. According to Sissoko and coworkers, age over 45 years, severe initial joint pain and underlying osteoarthritis are independent indicators of non recovery [8]. These findings are consistent with those reported in the seminal descriptions of CHIK-R [20,21], or found in recent case series [22,23], which showed a correlation between the age at disease onset and the duration of rheumatic symptoms.

Recently, we conducted the TELECHIK cohort study with the aim to address the topics of the burden of Chikungunya and perceived morbidity in the community after the 2005 to 2006 outbreak [24]. In this context, we performed this ancillary study to search for prognostic factors of long-lasting rheumatic musculoskeletal pain (RMSP) indicative of CHIK-R. We took the opportunity of this research to test several hypotheses with regard to a putative involvement of the host immune response in the pathomechanism of CHIK-R. Long-lasting RMSP could be driven by either the initial viral load or by the intensity of viral exposure under the challenge of repeated infective bites and waves of CHIKV in La Réunion, or by ongoing antigenic stimulation due to the persistence of CHIKV in host sanctuaries, using the value of CHIKV-specific IgG antibody titre measured at plateau phase, at the time of the seroprevalence study [25], as a proxy.

## Materials and methods

### Setting and design

La Réunion is a French overseas department of 787,836 inhabitants (Insee census 2006), located on a volcanic island in the south-western part of the Indian Ocean. During the years 2005 to 2006, 300,000 of the inhabitants (38.2%) were considered to have been infected [25].

**Figure 1:**
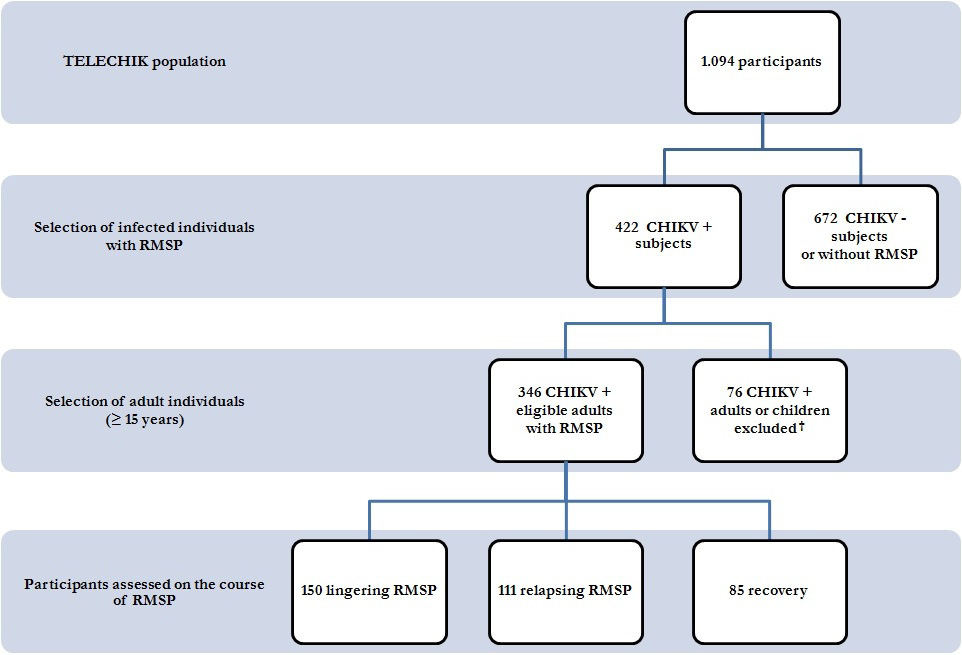
**Prognostic survey participation profile**. RMSP, rheumatic musculoskeletal pain. The prognostic survey cohort sample was issued from the TELECHIK survey, a population-based cohort study (November 2007 to May 2008) (23). After elimination of 582 Chikungunya virus (CHIKV)-seronegative subjects and 90 subjects not presenting with RMSP at disease onset, 76 subjects were excluded (50 children, 15 adults for relocations and absence of contact, 11 for the absence of overt temporality in the clinical course of Chikungunya rheumatism), leaving 346 eligible adult participants assessed on the course of RMSP. This population was shifted towards the selection of more women and older participants.

This study involved a subsample of the SEROCHIK and TELECHIK studies. Both studies have been reviewed extensively elsewhere [24,25]. The frame of the participant selection is given in Figure 1 and Additional file 1. Seroprevalence estimates have been measured on average 7.6 months (range 1 to 18 months) following acute infection for each individual, which time point was assumed to correspond to the plateau phase of the production of individual-specific-IgG and to the crucial time of evolvement to either recovery or the chronic phase. The TELECHIK study has been conducted in the framework of the SEROCHIK study between December 2007 and June 2008, on average eighteen months after the fall of the outbreak. In this study, the exposure to CHIKV was confirmed by CHIKV-specific IgG ELISA antibodies [24].

### Study population

The present study is a prognostic survey of the predictors of CHIK-R from the TELECHIK cohort [24]. Thus, it included all CHIKV seropositive adult participants (age ≥ 15 years) enrolled in the TELECHIK cohort, who had reported RMSP at disease onset.

### Data collection and follow-up

Participants were interviewed by telephone. The questionnaire was administered by one investigator, blind to the serological status, to ensure the best possible reproducibility [24]. It was composed of closed questions addressing the course of rheumatic manifestations in three consecutive time points (disease onset, one concomitant to the SEROCHIK study, and one for the TELECHIK inquiry). At disease onset, the questionnaire aimed to define the location of joint pain (hand, wrist, elbow, shoulder, neck, upper back, lower back, hip, knee, ankle, foot). At the TELECHIK time point, the questionnaire aimed to assess the clinical outcome, which was subsequently classified as recovery, relapsing, or lingering RMSP. Data on demographics, health status, knowledge of CHIKV transmission, habitat and environment were gathered from the SEROCHIK study [26].

Severe initial rheumatic involvement was defined as joint or muscle pain with fever and six or more painful sites among those listed above, and at least four other symptoms among the following: rash, headache, fatigue, mood or digestive disorders. Low to moderate rheumatic involvement was defined as RMSP with at most two of the preceding conditions.

Underlying comorbidities accounted for pre-existing metabolic syndrome (MetS), rheumatic disorders, and other chronic conditions (asthma, chronic obstructive pulmonary disease and cancer) [24]. Rheumatic disorders included IRDs, that is, RA, ankylosing spondylitis (AS), PsA (axial PsA, or PsA with peripheral joint involvement), hip, knee and hand osteoarthritis, and gout [12-14]. MetS was defined by at least one of the following conditions: body mass index ≥ 30 kg/m^2^, type-2 diabetes mellitus, hypertension, or any type of dyslipidaemia. The individual exposure level was generated by the result of a scoring system deriving a probability of infection that was assumed to highlight the infection load. The characteristics of this scoring system of CHIKV exposure are depicted in the footnote of Additional file 2.

The values of total CHIKV-specific IgG antibodies were the ones provided by the SEROCHIK study. On this behalf, the option was assumed that post-epidemic IgG titre measured at the plateau phase might be an accurate proxy for the exposure level within successive immune boosts following repeated *Ae. albopictus *infective bites throughout the epidemic.

### Laboratory methods

Total CHIKV-specific IgG antibodies were detected by direct ELISA, as established at the National Reference Centre (Pasteur Institute, Lyon, France). The technique was automated in an ETIMax 3000^® ^apparatus (DiaSorin, Rome, Italy). Optical density thresholds were calculated from a series of 30 negative sera collected in 2004, before the epidemic [27].

### Outcome measure

Recovery was defined by RMSP at disease onset, absent at the two last follow up time points. CHIK-R was defined by long-lasting RMSP (> 3 months), relapsing RMSP (present at one to two time points with impossibility of a conclusion of recovery), or lingering RMSP (present at three time points), irrespective of pain and health-related quality of life [28].

### Statistical analysis

Averages of age and CHIKV-specific IgG titres were compared between outcome categories using the Kruskal-Wallis and Cuzick tests, as appropriate. Predictors of long-lasting relapsing or lingering RMSP related to explanatory variables were assessed in bivariate analysis using recovery as the referent category. Weighted cumulative incidence rates (CIR) of RMSP were compared between categories of predictive variables using survey-adjusted Wald χ^2 ^tests to account for the sampling scheme (1: probability of inclusion as sampling weight). Design-based crude odds ratios (ORs) and 95% CI were determined for each modality of significant variables and overall *P*-values were calculated using the χ^2 ^likelihood ratio test.

At the first step of multivariate analysis, we fitted a full multinomial logistic regression model with age, gender, underlying comorbidity, exposure score, initial rheumatic involvement and CHIKV-specific IgG titres as explanatory variables, and relapsing or lingering RMSP as outcome variables with recovery as the referent category. From these covariates, we used a backward stepwise selection procedure to drop out non significant variables (output if *P *> 0.05). At the second step, we built in a minimal multinomial logistic regression model with all significant covariates from the precedent model plus gender. Indeed, we decided to force the gender because women are usually more susceptible to multiple musculoskeletal conditions [29]. At the third step, in a sensitivity analysis we built up a Poisson regression model without an option for robust calculation of variance, predictive of lingering RMSP with the same covariates to better assess the incidence risk ratio (IRR) of the most pejorative outcome with recovery as reference. Independent predictors of CHIKV-specific IgG titres were subsequently searched using multiple linear regression analysis. Statistical significance was set at *P *= 0.05; analysis was performed using Stata 10^® ^(StataCorp. 2008, Texas, USA).

### Ethical considerations

The TELECHIK study had received approval from the ethical committee for studies with human subjects (CPP) of Bordeaux and the National Commission for Informatics and Liberty (CNIL), as ancillary research completing the SEROCHIK study [24,28].

## Results

Among the 422 subjects included, 50 children (age < 15 years) and 26 adults were ruled out due to fear of poorly characterizing the course of CHIK-R, as well as to enhance benchmarking with some of the rare articles published in the field [6-8,18-21,30,31]. The study participation profile is displayed in Figure 1.

Three hundred and forty-six subjects were analyzed for the determinants of CHIK-R on average two years after infection (range 15 to 34 months). The median age was 50 years (range 15 to 91 years). The sex ratio of men to women was 0.61 (131 to 215). All participants were assessed for the CHIK-V-specific IgG value on an average of 232 days (7.6 mo) after acute infection (range 1 to 18 mo).

The characteristics and the localizations of CHIK-R at disease onset in this population are presented in Table 1. In bivariate analysis, women tended to report lingering RMSP more often than men (42.2% vs 31.9%, *P *= 0.083). The intensity of the CHIK-R outcome increased with age, from the recovery, passing through the relapsing, to the lingering group (*P *< 0.001). Participants with an underlying comorbidity were older than those without. Among the latter, those with a pre-existent rheumatic disorder were the oldest. Body mass index and previous rheumatic disorders were not linked to CHIK-R outcome. The subjects with two or more comorbidities were more likely to declare chronic RMSP than those without an underlying condition. The presence of MetS was associated with both relapsing (crude OR 2.31, 95% CI 1.04, 5.12) and lingering RMSP (crude OR 3.93, 95% CI 1.35, 8.35) although this link was skewed by adjustment for age (data not shown). Among the criteria defining MetS, type-2 diabetes mellitus and dyslipidaemia were linked irrespectively to the expression of CHIK-R, whereas arterial hypertension was associated restrictively with lingering RMSP. Renal failure was also associated with CHIK-R outcome.

**Table 1:** Characteristics of 346 CHIKV-infected subjects ≥ 15 years of age assessed for Chikungunya rheumatism in the TELECHIK study, La Réunion, 2007 to 2008

Characteristics	Number	Unweighted* proportion, %	Weighted** proportion, %
**Gender**			
Male	131	37.9	37.9
Female	215	62.1	62.1
**Age**			
15 to 29 yrs	59	17.1	21.4
30 to 44 yrs	90	26.0	33.3
45 to 59 yrs	97	28.0	26.5
≥ 60 yrs	100	28.9	18.8
**Body mass index**			
< 25.0 kg/m^2^	182	52.9	51.1
25.0 to 29.9 kg/m^2^	119	34.6	34.9
≥ 30.0 kg/m^2^	43	12.5	13.9
**Sites of arthralgia at disease onset**			
Hand	262	75.7	76.9
Wrist	254	73.4	74.0
Elbow	156	45.1	52.3
Shoulder	195	56.4	57.0
Neck	165	47.7	46.9
Upper back	160	46.2	45.1
Lower back	163	47.1	47.9
Hip	95	27.5	27.6
Knee	234	67.6	66.9
Ankle	259	74.9	76.3
Foot	253	73.1	73.9
**Sites of arthralgia at disease onset, n**			
1	21	6.1	5.3
2 to 5	118	34.1	33.8
≥ 6	207	59.8	60.0

*Percentages are calculated roughly in the subsample of the 346 analyzed subjects; **percentages are calculated taking into account the multistage sampling scheme.

Exposure score was not related to CHIK-R, making obsolete the hypothesis of a relationship between recurrent exposures and the expression of RMSP. On the other hand, there was a significant non linear positive association between CHIKV-specific IgG titres and the expression of CHIK-R. Thus, CHIKV-specific IgG level averages increased from the recovery, passing through the relapsing, to the lingering group (*P *< 0.001).

In a full multinomial logistic regression model (Table 2) controlling the exposure level, gender and underlying comorbidities (MetS, history of IRDs and other chronic conditions), the predictors of relapsing RMSP were age ≥ 60 years, severe initial rheumatic involvement, and increasing CHIKV-specific IgG titres. Prognostic factors for lingering RMSP were age ≥ 45 years (increasing OR from the group aged 45 to 59 years group to the group aged ≥ 60 years), severe initial rheumatic involvement and increasing CHIKV-specific IgG titres.

**Table 2:** Predictors of Chikungunya rheumatism issues in subjects ≥ 15 years of age (full model) in the TELECHIK study, La Réunion, 2007 to 2008

Outcomes (versus recovery as reference)	Relapsing rheumatic musculoskeletal pain					Lingering rheumatic musculoskeletal pain				
**Determinants**	**N**	**CIR, %**	**Adjusted odds ratio**	**95% CI**	***P*- value**	**N**	**CIR, %**	**Adjusted odds ratio**	**95% CI**	***P*- value**

**Gender**					0.536					0.562
Male	41	34.7	1			48	31.9	1		
Female	70	32.4	0.78	0.34, 1.74		102	42.2	0.78	0.33, 1.82	
**Age**					0.013					< 0.001
15 to 29 yrs	19	27.3	1			12	20.7	1		
30 to 44 yrs	32	37.9	1.60	0.57, 4.51		28	30.4	1.64	0.51, 5.31	
45 to 59 yrs	27	30.0	2.67	0.78 - 9.10		52	50.2	5.62	1.52, 20.79	
≥ 60 yrs	33	36.3	9.20	2.31, 36.59		58	55.6	16.03	3.82, 67.13	
**Underlying comorbidity**					0.667					0.555
None	56	32.8	1			62	32.5	1		
Metabolic syndrome without rheumatic disorders*	35	31.8	1.45	0.45, 4.65		63	53.6	1.92	0.59, 6.24	
Rheumatic disorders with or without metabolic syndrome**	6	33.5	1.67	0.20, 13.53		16	53.8	2.11	0.27, 16.26	
Other conditions^¶^	12	44.7	2.02	0.62, 6.49		8	18.8	2.02	0.60, 6.81	
**Initial rheumatic involvement**					0.008					0.001
Low to moderate^† ^	70	31.8	1			77	31.0	1		
Severe^‡^	41	35.6	3.52	1.40, 8.87		73	50.0	5.43	1.95, 15.11	
**CHIKV-specific IgG titre **(per one unit increase)	111	33.2	3.31	1.82, 5.99	< 0.001	150	38.3	5.72	2.74, 12.51	< 0.001
**Exposure Score^#^**					0.132					0.195
First quartile class (-2.526; -0.415)	21	32.3	1			32	38.7	1		
Second quartile class (-0.400; 0.018)	27	31.9	0.60	0.20, 1.73		33	33.2	0.48	0.16, 1.42	
Third quartile class (0.026; 0.432)	23	27.0	0.53	0.17, 1.70		35	41.7	0.70	0.22, 2.13	
Fourth quartile class (0.433; 1.435)	25	38.3	2.09	0.57, 7.59		39	42.6	1.87	0.43, 7.97	

CIR, cumulative incidence rate; CHIKV, Chikungunya virus; N, number. All estimations and *P*-values are weighted by the sampling scheme. *Metabolic syndrome was defined by any of the following features: body mass index ≥ 30 kg/m^2 ^, diabetes mellitus, arterial hypertension, or any type of dyslepidemia; **hip, knee or hand osteoarthritis, rheumatoid arthritis, ankylosing spondylitis and psoriatic spondylarthritis and other types of rheumatic disorders, such as gout; ^¶^coronary heart disease, asthma, chronic bronchitis, renal insufficiency, cancer; **^#^**exposure score build up with six covariates and two derivatives (eight components) including residence area, housing type, a first interaction term between the residence area and housing type, deciles of altitude, household size, history of recent Chikungunya-related picture in the neighbours, a second interaction term between the household size and history of Chikungunya, and last, the answer to the question: 'Is Chikungunya virus a mosquito-borne virus?'. All subjects had arthralgia or muscle pain at disease onset. ^†^Joint or muscle pain with at most two of the three following conditions: fever, six or more localizations of arthralgia, at least four other symptoms; ^‡^fever and six or more localizations of arthralgia and at least four other symptoms.

The key predictors of relapsing or lingering RMSP were identified in the minimal multinomial logistic regression model adjusted for gender after dropping out the exposure score and underlying comorbidities. As a result, independent factors were age ≥ 45 years (with increasing OR from the group aged 45 to 59 years group to the group aged ≥ 60 years), severe initial rheumatic involvement and increasing CHIKV-specific IgG titres (Table 3). Of note, the OR of the predictors of CHIK-R increased with the intensity of CHIK-R (higher for lingering than for relapsing RMSP). The same conclusion was reached taking relapsing RMSP plus recovery as the reference (Additional file 3).

**Table 3:** Predictors of Chikungunya rheumatism issues in subjects ≥ 15 years (final model), TELECHIK study, La Réunion, 2007-2008

Outcomes (versus recovery as reference)	Relapsing rheumatic musculoskeletal pain			Lingering rheumatic musculoskeletal pain		
**Determinants**	**Adjusted OR**	**95% CI**	***P*-value**	**Adjusted OR**	**95% CI**	***P*-value**

**Gender**						
Male	1			1		
Female	0.92	0.44, 1.92	0.831	1.03	0.45, 2.31	0.951
**Age**						
15 to 29 yrs	1			1		
30 to 44 yrs	2.08	0.81, 5.32	0.124	2.29	0.74, 6.99	0.146
45 to 59 yrs	2.93	1.00, 8.57	0.049	6.35	1.82, 22.10	0.004
≥ 60 yrs	10.44	3.50, 31.10	< 0.001	22.30	6.36, 78.06	< 0.001
**Initial rheumatic involvement**						
Low to moderate^† ^	1			1		
Severe^‡^	3.60	1.58, 8.21	0.002	5.52	2.20, 13.81	< 0.001
**CHIKV-specific IgG titre **(per one unit increase)	3.16	1.82, 5.48	< 0.001	6.16	2.88, 13.20	< 0.001

OR, odds ratio; CHIKV, Chikungunya virus. All estimations and *P*-values are weighted by the sampling scheme. All subjects had joint or muscle pain at disease onset. ^†^Joint or muscle pain with at most two of the three following conditions: fever, six or more localisations of arthralgias, or at least four other symptoms; ^‡^fever and six or more localizations of arthralgias and at least four other symptoms.

**Figure 2:**
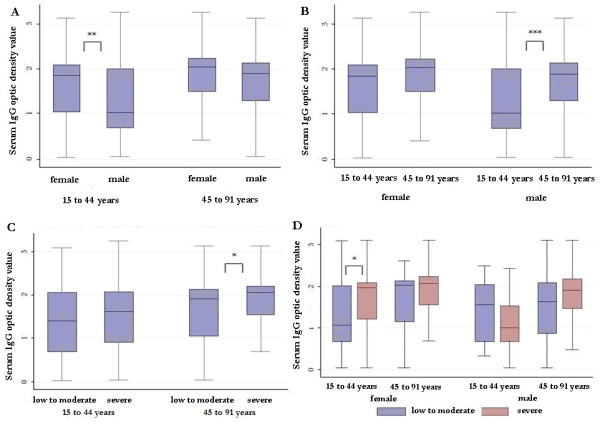
**Stratification of Chikungunya virus (CHIKV)-specific IgG titre levels according to explanatory variables in the TELECHIK study, La Réunion, 2007 to 2008**. (CHIKV)-specific IgG titre levels are shown by gender in the two age groups (A), by age in according to gender (B), by severity of presentation in the two age groups (C), and by severity of presentation by age group and gender (D). Boxes represent medians and interquartile (Q_1_-Q_3_) ranges, whiskers represent minimal and maximal values. Optic density values were compared using Mann-Whitney tests: *P < 0.05; **P < 0.01; ***P < 0.001.

Interestingly, CHIKV-specific IgG titres were linked to female gender and correlated with age but not with the probability of infection (Table 4). The highest sex-related difference in IgG levels was observed in the reproductive age group (Figure 2a). Concurrently, the highest age difference was recorded in male individuals (Figure 2b). The CHIKV-specific IgG titre values also correlated with the intensity of initial rheumatic involvement through an age-dependent gradation that reached significance in the oldest subjects (Figure 2c). However, in detail, a severe clinical picture contributed to increasing IgG titres only in young women (Figure 2d).

**Table 4:** Predictors of Chikungunya virus-specific IgG titres in subjects ≥ 15 years of age in the TELECHIK study, La Réunion, 2007 to 2008

Full multiple regression linear model of CHIKV-specific IgG titres (*n *= 309)				
**Variable**	**β**	**SD**	**T**	***P-*value**

Gender	0.143	0.088	1.63	0.105
Age, yrs	0.005	0.002	2.36	0.019
Estimated probability of infection^*#*^	0.238	0.290	0.82	0.412
Initial rheumatic involvement	0.143	0.876	1.64	0.102
Time elapsed between infection and test^¶^	0.001	0.000	1.94	0.053
Constant	0.810	0.210	3.85	< 0.001

**Minimal multiple regression linear model of CHIKV-specific IgG titres (*n *= 344)**				

**Variable**	**β**	**SD**	**T**	***P-*value**

Gender	0.183	0.081	2.25	0.025
Age, yrs	0.006	0.002	2.82	0.005
Initial rheumatic involvement	0.169	0.811	2.09	0.037
Constant	1.088	0.129	8.39	< 0.001

CHIKV, Chikungunya virus. ^#^Derived from the exposure score; ^‡^fever ± six or more localisations of arthralgias and at least four other symptoms (defined as severe if the three conditions are gathered, low to moderate if not); ^¶^time elapsed between onset of infection and ELISA test.

Thus, CHIKV-specific IgG titres correlated with the intensity of CHIK-R but not to the infection load, which argues against the notion that the rise of CHIKV-specific IgG titres may be due to re-infection following new introduction and waves of CHIKV in La Réunion. In another manner, the sex and age-related discrepancies observed across the different clinical pictures at presentation do not favor major involvement of the viral load in the pathomechanism of CHIK-R. We conclude that the host humoral response to the persistence of ongoing antigenic stimulation is a plausible clue to long-lasting RMSP.

## Discussion

Here we report a prognostic survey of CHIK-R based on the subset of participants reporting RMSP at the onset of CHIKV infection, who were extracted from the TELECHIK cohort study, a population based-study aimed at assessing the disease burden and perceived morbidity in La Réunion island community after the 2005 to 2006 CHIKV outbreak [24]. The major findings of our survey confirm the incidence at population level of CHIK-R across different variable categories, and the prognostic values of advanced age (≥ 45 years), severe initial rheumatic involvement at presentation, and rise in CHIKV-specific IgG titre through the recovery/chronic phases to predict the severity of CHIK-R over time. Thus, among previously uninfected individuals [25], the subjects ages 45 to 59 years and particularly the oldest subjects (≥ 60 years) were more likely to evolve towards relapsing and lingering chronic RMSP in an age-dependant dose-response manner. In addition, the risk estimate for severity of CHIK-R among the individuals in the 45-59 years age group was even increased towards the categories of outcome (that is, from the relapsing to the lingering feature group) in comparison with younger peers (aged 15 to 29 years). In the same way, the subjects who experienced severe initial rheumatic involvement (six or more painful sites with at least four other symptoms) at the acute stage of infection were more likely to exhibit chronic RMSP on follow up. Concurrently, the subjects who developed a strong CHIKV-specific IgG response shared a similar issue. These results were robust and were observed across different regression models controlling the exposure level to the infection (that is, the infection load) and major confounders such as female gender and underlying comorbidities. Likewise, these two factors have been previously recognized to be associated with poor health-related quality of life in people living with several musculoskeletal conditions, including IRDs, osteoarthritis, low back pain and soft tissue disorders, as reported in an Italian population [29].

It is noteworthy that in bivariate analysis female gender tended to be associated with lingering RMSP. Consistently, this finding has been previously reported for persistent arthralgias among CHIKV-infected subjects followed in the Italian cohort in the setting of the 2007 local CHIKV outbreak [19]. Of note, the strength of this association was modulated in our cohort by the level of the humoral immune response, which was found to be much higher among female than male patients from both the Réunion (*P *< 0.001) and Italian Chikungunya cohorts (Figure 2a). Such a gender difference in the CHIKV-specific IgG level was found at the baseline and may be due to the natural estrogen-driven shift towards the Th2 cytokine profile for promoting antibody production in women, or to the inhibitory effect of dihydrotestosterone (DHS) on B cell functioning in men [32]. On an other hand, it may also be due to higher production of immunosuppressive IL-10, which has been found to correlate positively with DHS and negatively with serum IgG_3 _titres in male patients with other viral infections [33,34]. These same mechanisms may have driven the discrepancy between IgG titres following CHIKV infection in men and women. Interestingly, the isotypic switch observed in Singapore CHIKV-infected patients referred exclusively to the IgG_3 _subclass [35]. Though we could not characterize the contribution of specific IgG isoforms, our results are consistent with those of the Italian cohort in which IL-10 levels decreased over time following the acute stage, in parallel with the rise in specific IgG level [36].

In the present study, more than 50% of the subjects ≥ 45 years of age at the time of infection still suffered from lingering RMSP on average two years after acute infection (Table 2). Other contributors have found a similar burden [6-8,22,23]. The prognostic value of advanced age as a harbinger of CHIK-R at disease onset has gained attention since the seminal description of CHIK-R [20,21]. Consistently, the positive link between age and the duration of RMSP has been confirmed in recent years by several cohort studies [6,8,19,23]. In line with these authors, we found a strong dose-response between increasing age over 45 years and the severity of CHIK-R, arguing for a causal relationship linking age to the duration and severity of CHIK-R. Moreover, a positive association between high titres of CHIKV-specific IgG in the plateau phase and long-lasting arthralgias has been observed contemporaneously from a pilot study by our group and in the Italian cohort [18,19]. A possible mechanistic hypothesis may be that an imbalance towards B cell expansion and differentiation, in response to IL-6 secretion following the progression of both immunosenescence [37] and Chikungunya [35,36,38], is triggered by viral persistence in host sanctuaries [15,17,18].

The contribution of underlying comorbidity has long been considered a prerequisite to the subsequent expression of CHIK-R [5-8,18-24,31,32], and a pre-existing rheumatic disorder was reported among 44% of the patients hospitalized after a CHIKV infection in La Réunion [7]. A history of arthralgia before the outbreak was associated with persistent joint pain in the Italian cohort [19]. Of the rheumatic disorders that could contribute to CHIK-R, RA [10,11,15], seronegative spondylarthritis [10,31], soft tissue rheumatism [11], osteoarthritis [7,8], and gout [8] have been reported to afflict CHIKV-infected patients. Previously, in the TELECHIK study, we were able to link osteoarthritis to long-lasting RMSP in the whole cohort [24], but failed to replicate this result in the subset of the arthralgic participants at disease onset. Indeed, rheumatic disorders were more likely to be associated with chronic RMSP when associated with one or more components of the MetS (that is, obesity, type-2 diabetes mellitus, hypertension or dyslipidaemia). However, this relationship did not resist to adjustment for age. Importantly, arterial hypertension and cumulative comorbidities have previously been associated with CHIK-R [8]. Therefore, a high incidence of lingering RMSP was observed in MetS-positive subjects (53.8%). On the one hand, CHIKV has been suggested to trigger auto-immune IRDs such as RA or PsA [11,12] and there is a growing body of evidence linking IRDs to MetS [39], especially RA or PsA [40]. On the other hand, undifferentiated arthralgias according to rheumatologic guidelines [12-14], which represent the hallmark of CHIK-R, should be considered for specific involvement in systemic inflammation and enhancement of MetS, assuming these two risk factors are key issues in the increased incidence of cardiovascular diseases occurring among IRD patients [40]. The issues should be considered of importance and deserve further studies to close a putative gap between Chikungunya and MetS.

The prognostic value of severe initial rheumatic involvement for predicting long-lasting RMSP is classical with alphaviruses, and encompasses most of the human pathogens included in the SFV serocomplex plus the Sindbis virus [1,41,42]. For CHIKV, it was first reported in La Réunion [8] and subsequently confirmed in other settings [19,22,23,30]. The intensity of symptoms at disease onset has been linked to plasma viral load, CRP level, and Th1-driven cytokine traffic [3,15,41-44]. Indeed, in our study, a positive link between the intensity of symptoms at disease onset and the course of CHIK-R was found beyond the distribution of CHIKV-specific IgG titre values (Tables 2 and 3, and Additional file 3). Indeed, the relationship was found independent of IgG levels, while observing a correlation between the severity of the clinical picture and the IgG level (Table 4), highlighting a proper role of each of these factors on the outcome. This relationship may involve the pivotal action of IL-6 in the recovery phase of infection [35,36,38], which favours the switch from innate to adaptive immunity leading to B cell expansion and IgG_3 _production [35]. Although controversial, IL-6 secretion at the acute stage may also explain the concomitant increase in CRP level [5,43] and its predictive value for CHIK-R [15,31].

In the absence of evidence of early-onset autoimmunity or circulating immune complexes [15], the proper role of IgG on CHIK-R remains elusive. From our study, the fact that non-linearity characterizes the positive association between CHIK-R and specific IgG levels argues for the contribution of other factors. The possibility of mixed cryoglobulinemia has been suggested [45] but not replicated in observational studies [18]. Long-term persistence of IgM has been reported with CHIKV [27], sometimes associated with erosive arthritis [46], so that it is the timing of antibody commutation rather its significance (putative persistence of CHIKV antigens) that would be of critical importance.

We could not draw insights from the absence of a link between the exposure level and the course of CHIK-R, or between the infection load and CHIK-specific IgG titres. This could be due to the fact that our exposure scoring system did not capture the host behavior after infection throughout the epidemic, or that of the risk of being infected changed over time due to vector control measures. With the potential contribution of memory B cells to the level of CHIKV-specific IgG titres and subsequent related immunopathology, this negative finding does not imply failure to take care to prevent the burden of CHIK-R over the course of long-lasting CHIKV outbreaks. Indeed, the people at risk for CHIK-R should now be targeted using anti-*Ae. albopictus *saliva IgG abs [47].

This study has some strengths and limitations. The limitations inherent in the TELECHIK study have been reviewed extensively elsewhere [24]. Regarding the rheumatologic aspects of Chikungunya, one of the main drawbacks to inference would be that in a declarative survey, patients were not examined physically by a clinician to rule out differential diagnoses and confirm the incapacity. However, we had previously demonstrated that the role of subjectivity was negligible in the TELECHIK study [24]. Here, we have shown that the spectrum of early clinical manifestations declared at disease onset are slightly overestimated compared to those described in the setting of medicine or rheumatology clinics [5,6]. This discrepancy could be explained in our study by the selection of more female and older participants, highly susceptible to CHIK-R, as well as by the enlargement of the window of disease onset due to the retrospective assessment of complaints, rather than to significant recall bias. Indeed, clinicians may also be inattentive to subtle complaints in the context of being overwhelmed by the number of consultations during epidemics. In that case, we also demonstrate in another sensitivity analysis that our findings are robust and valid for the most pejorative expression of CHIK-R (Additional file 3). Thus, we are confident that declaration bias was unlikely to have changed the scope of our findings.

## Conclusions

The TELECHIK cohort study provides valuable clues in the field of rheumatology by contributing to strengthen the recognition and interpretation of the predictors of CHIK-R. Our findings also demonstrate the putative prognostic value of the IgG host level assessed in the recovery/chronic phase times. Thus, the standardized assessment of the engagement of the humoral response and the timing of production of neutralizing IgG_3 _might help to identify CHIKV patients at risk for long-lasting RMSP. This issue might also change the paradigm from almost exclusive interest in the innate immune response to adaptive immune trafficking, and thus pave the way to new avenues of research into Chikungunya [35,36]. Consistently, these perspectives should focus on an integrative approach of exploring the timing of the IgG switch and B cell signalling, innate/adaptive collaborations, chronic infection and aging [37], or potential roads to late-onset autoimmunity [48] and lymphoproliferative disorders [49].

## Abbreviations

abs: antibodies; AS: ankylosing spondylitis; CHIK-R: Chikungunya rheumatism; CHIKV: Chikungunya virus; CIR: cumulative incidence rate; CNIL: Commission Nationale Informatique et Libertés; CPP: Comité de Protection des Personnes; CRP: C-reactive protein; DHS: dihydrotestosterone; ELISA: enzyme-linked immunosorbent assay; ESR: erythrocyte sedimentation rate; HLA: human leucocyte antigen; IFN: interferon; IgG: immunoglobulin G; IL: interleukin; Insee: Institut National des Statistiques et des Etudes Economiques; IRDs: inflammatory rheumatic disorders; IRR: incidence risk ratio; MetS: metabolic syndrome; OR: odds ratio; PsA: psoriatic arthritis; RA: rheumatoid arthritis; RMSP: rheumatic musculoskeletal pain; RRV: Ross River virus; SFV: Semliki Forest virus.

## Competing interests

The authors declare that they have no competing interests.

## Authors' contributions

PG1 analyzed the data, drafted and reviewed the manuscript; AF helped to design the study, analyzed the data, reviewed the data for consistency and errors, as also the manuscript; AM, EB, GB1, BAG, DM, GB2 and FF reviewed the manuscript, each in his field of expertise for consistency and perspectives; CM performed telephone interviews, and entered the data in the computer file; KB performed data management; OR helped to analyze the data; AM, PG2 and SK performed serologies; FF was the principal investigator of the SEROCHIK and TELECHIK surveys. All authors read and approved the final manuscript.

## Supplementary Material

Additional file 1 **Figure showing the TELECHIK study participation profile**. The TELECHIK cohort sample was issued from the cross-sectional SEROCHIK survey, a population-based seroprevalence study held between 17 August and 20 October 2006, involving a random sample of the Réunion island community (2,442 individuals), selected by the French National Institute for Statistics and Economical studies (Insee) after stratification by age, gender, residence area, municipality size, and housing type [24,25]. The selection procedure of the TELECHIK population considered six exposure strata: true positive (symptomatic CHIKV infection), false negative (asymptomatic infection), not knowing positive (infection without memory of symptoms and serostatus), true negative (asymptomatic CHIKV negative to infection), false positive (symptomatic CHIKV negative to infection), and not knowing negative (absence of infection without memory of symptoms and serostatus), in order to account for declaration bias and the representativeness of the cohort, taking into account a feasibility constraint. Two subsets of the same size of true positives and true negatives were selected after stratification by age, gender and area of residence to control repartition bias, the allocation of participants within the six strata being conducted by applying reasoned sampling fractions (true positive 0.7 and true negative 0.46), or systematic selection (false positive, not knowing positive, false negative, not knowing negative). After elimination of those missing the call or individuals refusing, exclusion of another 54 individuals because of incomplete data or mismatched responders (different from the index person, parents, or legal guardian)*, the population was slightly skewed towards the selection of more women and older participants [23]. Click here for file

Additional file 2 **Figure showing performances of the exposure scoring system in predicting Chikungunya virus infection in the development population (*n *= 1,863) in the SEROCHIK study, 2006**. The exposure score was built up with six covariates and two derivatives (eight components) including residence area (north, west, south, east), housing type (collective/individual), a first interaction term between residence area and housing type, deciles of altitude, household size (1, 2 to 4, ≥ 5 persons), history of recent Chikungunya-related picture in the neighbours (no/yes/don't know), a second interaction term between the household size and history of Chikungunya, and last, the answer to the question: 'Is Chikungunya virus a mosquito-borne virus (no/yes)?'. It was developed from a population of 2,101 eligible adult individuals (≥ 15 years of age) enrolled in the SEROCHIK survey. After elimination of 238 individuals (11.3%) due to missing data, the score displayed a range of 320 eigen values in 1,863 individuals according to a continuous multimodal distribution. The discrimination (or the ability to distinguish infected from uninfected individuals) and calibration (or the adequation between predicted and observed infections over a range of probabilities) performances of the exposure scoring system were considered both satisfactory in the development population (receiver operator characteristic area or A*z *index: 0.70, 95% CI 0.67, 0.72; goodness of fit *F*-adjusted test, *P *= 0.840). Click here for file

Additional file 3 **Table showing sensitivity analysis predicting lingering rheumatic musculoskeletal pain in subjects ≥ 15 years of age in a Poisson regression model in the TELECHIK survey, La Réunion, 2007 to 2008**. Click here for file
