# Supplementary figures and images for: Predictors of Chikungunya rheumatism: a prognostic survey ancillary to the TELECHIK cohort study
Source: Arthritis Res Ther. 2013 Jan 9;15(1):R9. doi: 10.1186/ar4137 (PMC3672753; doi:10.1186/ar4137)

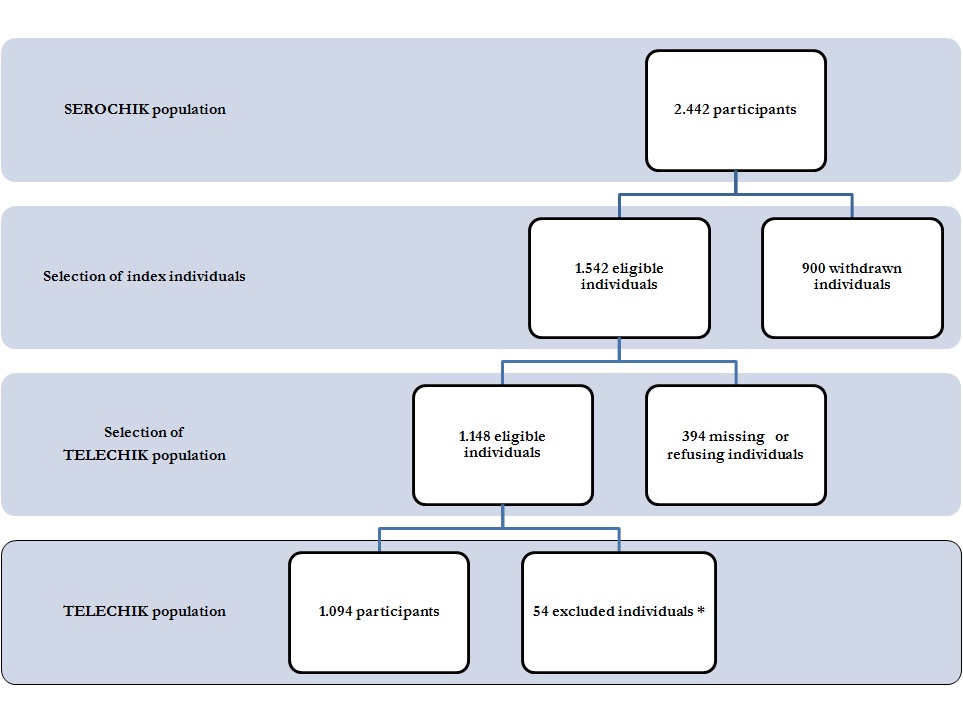

Supplement: Additional file 1 — Figure showing the TELECHIK study participation profile. The TELECHIK cohort sample was issued from the cross-sectional SEROCHIK survey, a population-based seroprevalence study held between 17 August and 20 October 2006, involving a random sample of the Réunion island community (2,442 individuals), selected by the French National Institute for Statistics and Economical studies (Insee) after stratification by age, gender, residence area, municipality size, and housing type [24,25]. The selection procedure of the TELECHIK population considered six exposure strata: true positive (symptomatic CHIKV infection), false negative (asymptomatic infection), not knowing positive (infection without memory of symptoms and serostatus), true negative (asymptomatic CHIKV negative to infection), false positive (symptomatic CHIKV negative to infection), and not knowing negative (absence of infection without memory of symptoms and serostatus), in order to account for declaration bias and the representativeness of the cohort, taking into account a feasibility constraint. Two subsets of the same size of true positives and true negatives were selected after stratification by age, gender and area of residence to control repartition bias, the allocation of participants within the six strata being conducted by applying reasoned sampling fractions (true positive 0.7 and true negative 0.46), or systematic selection (false positive, not knowing positive, false negative, not knowing negative). After elimination of those missing the call or individuals refusing, exclusion of another 54 individuals because of incomplete data or mismatched responders (different from the index person, parents, or legal guardian)*, the population was slightly skewed towards the selection of more women and older participants [23]. [file ar4137-S1.JPEG]

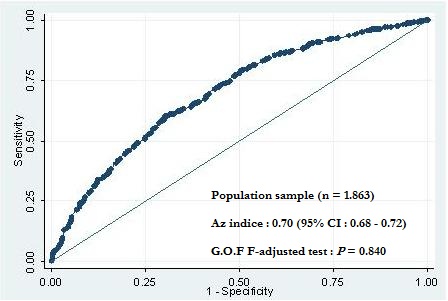

Supplement: Additional file 2 — Figure showing performances of the exposure scoring system in predicting Chikungunya virus infection in the development population (n = 1,863) in the SEROCHIK study, 2006. The exposure score was built up with six covariates and two derivatives (eight components) including residence area (north, west, south, east), housing type (collective/individual), a first interaction term between residence area and housing type, deciles of altitude, household size (1, 2 to 4, ≥ 5 persons), history of recent Chikungunya-related picture in the neighbours (no/yes/don't know), a second interaction term between the household size and history of Chikungunya, and last, the answer to the question: 'Is Chikungunya virus a mosquito-borne virus (no/yes)?'. It was developed from a population of 2,101 eligible adult individuals (≥ 15 years of age) enrolled in the SEROCHIK survey. After elimination of 238 individuals (11.3%) due to missing data, the score displayed a range of 320 eigen values in 1,863 individuals according to a continuous multimodal distribution. The discrimination (or the ability to distinguish infected from uninfected individuals) and calibration (or the adequation between predicted and observed infections over a range of probabilities) performances of the exposure scoring system were considered both satisfactory in the development population (receiver operator characteristic area or Az index: 0.70, 95% CI 0.67, 0.72; goodness of fit F-adjusted test, P = 0.840). [file ar4137-S2.JPEG]
