# Supplementary material for: Predictors of Chikungunya rheumatism: a prognostic survey ancillary to the TELECHIK cohort study
Source: Arthritis Res Ther. 2013 Jan 9;15(1):R9. doi: 10.1186/ar4137 (PMC3672753; doi:10.1186/ar4137)
Supplement: Additional file 3 — Table showing sensitivity analysis predicting lingering rheumatic musculoskeletal pain in subjects ≥ 15 years of age in a Poisson regression model in the TELECHIK survey, La Réunion, 2007 to 2008. [file ar4137-S3.DOC]

| **Table S1. Sensitivity analysis predicting lingering rheumatic musculoskeletal pain in subjects ≥ 15 years, TELECHIK survey, La Réunion, 2007-2008** | | | |
| --- | --- | --- | --- |
| **Outcome (vs recovery or relapsing RMSP)** | **Lingering rheumatic musculoskeletal pain** | | |
| **Determinants** | **Adjusted IRR** | **95% CI** | ***P value*** |
| **Gender** |  |  |  |
| Male | 1 |  |  |
| Female | 1.15 | 0.83 – 1.58 | 0.393 |
| **Age** |  |  |  |
| 15-29 yrs | 1 |  |  |
| 30-44 yrs | 1.51 | 0.81 – 2.82 | 0.004 |
| 45-59 yrs | 2.33 | 1.31 – 4.14 |  |
| ≥ 60 yrs | 2.39 | 1.35 – 4.20 |  |
| **Initial rheumatic involvement** |  |  |  |
| Low to moderate ^†^ | 1 |  |  |
| Severe ^‡^ | 1.42 | 1.06 - 1.90 | 0.018 |
| **CHIKV-specific IgG titre** |  |  |  |
| 0.0280 - 1.0415 (Q_1_) | 1 |  |  |
| 1.0416 - 1.8230 (Q_2_) | 0.55 | 0.29 – 1.05 |  |
| 1.8240 - 2.1300 (Q_3_) | 1.41 | 0.85 – 2.33 | < 0.001 |
| 2.1310 - 3.2480 (Q_4_) | 2.09 | 1.32 – 3.30 |  |
| RMSP: rheumatic musculoskeletal pain. Adjusted Incidence Risk Ratios (IRR) and 95% confidence interval (95%CI) were calculated taking recovery plus relapsing RMSP as reference. All estimations and *P* *values* are weighted by the sampling scheme. All subjects had joint or muscle pain at disease onset.  ^†^ Joint or muscle pain with at most two of the three following conditions: fever, six or more localisations of arthralgias, or at least four other symptoms. ^‡^ fever and 6 or more localisations of arthralgias and at least 4 other symptoms. | | | |
